# Sustainable Traditional Medicine: Taking the Inspirations from Ancient Veterinary Science

**DOI:** 10.1093/ecam/nen071

**Published:** 2010-10-20

**Authors:** Sanjeev Rastogi, Krishna Kaphle

**Affiliations:** ^1^State Ayurvedic College & Hospital, India; ^2^Department of Holistic Medicine, BMCRC, Vatsala Hospital, Tulsi Das Marg, Lucknow, India; ^3^Veterinary Teaching Hospital, IAAS, Rampur, Chitwan, Nepal

## Abstract

Rapid reduction in natural resources as a consequence to the expanded urbanization, global warming and reduced natural habitat posed a considerable threat to the sustainability of traditional medicine. Being completely dependent upon natural resources like herbs, minerals and animal products, traditional medicine would possibly rank first in order of extinction of heritage if an alternative way is not considered well in time. In reference to the use of animal products, Ayurveda presents some unique examples where animals are used without causing harm to them and so without posing a threat to their existence. In the current context, when natural resources are facing a threat to their existence, a revisit to these ideas may give us a new insight to refine our look at natural resources used in traditional medicine.

## 1. Need of Sustainability in Traditional Medicine: The Rationale

“It became my ambition to link the knowledge of Traditional Chinese Medicine (TCM) and Ayurveda from the Indian subcontinent and their integration with other systems of medicine, including Western medicine (WM), to achieve the concept of Sustainable Medicine, firstly for animals and then for humans" this statement of Kaphle et al. [1] appeared in a recent issue of *eCAM* has a potential to be elaborated on grounds of better utilization of veterinary science for bringing forth the concept of sustainable Traditional medicine (TM) for human with added ethical advantages. Cooper [2] further elucidated the idea by saying that animals can become the focus of TCM (of TM in general) approaches to disease rather than the exact opposite, that is, using their parts to cure diseases of human.

The steep rising pattern of demand and consequent growth in Complementary and Alternative Medicine (CAM) sector has led to some pertinent situations. Ayurveda and other traditional medical systems are facing the problem of nonavailability of genuine raw material for past many decades [3]. Ayurveda some how tried to meet the demands by seeking alternatives for the conventional herbs or other ingredients, which are either not available or have lost their identity. This small step however could not meet efficiently to the recent spurt in the demand of TM. Over exploitation, changes in global environment and loss of delicate ecosystems due to extensive urbanization collectively resulted in convergence of gene pool, evolution of poorly endurable individuals and consequent loss of many species, which have been prevalent in early recent days. The consequences are more alarming than it may superficially appear. The future TM has a possibility to become even more expensive than the conventional medicine of today if its resources are not made easily available or accessible. Demand-oriented market pressures and poor quality checks in TM has motivated its producers to go for mass productions without taking care for the finer details of plant cultivation and their utilization as described traditionally. We may have gone a long way in thinking of how to preserve the biodiversity and gene pools, but this does not seem to have a direct effect upon the community who is simply looking for a traditional remedy for their ailment which they believe as to be helpful. Story of the animals taking care of our health is no different. The stunning developments and advances in the field of medicine made in past many decades also have an associated brutal history and as a consequence our health assurance and sustainability has been made dependent upon the lives of innocent animals. Modern medical advances are completely dependent upon exploratory and experimental studies involving pity animals. TMs are no different as they unknowingly encourage to unethical animal killing for their products of proven or unproven medicinal properties. At both places this have been questioned, objected and asked to look for a better and effective alternative to continue the researches and also to sustain the TM. We need to become proactive by looking at future perspectives of TM in view of its current growth pattern. The simple question, which emerges out of this discussion, is that how we would be meeting the demands for plants and animal products needed to fulfill the future requirement if a current trend of global entrustment of CAM continues. The question is rather difficult to answer and this is why a need to think for sustainable TM arise. An over view of Ayurveda in regards to its vision for animals and their possible utilization for human welfare without harming them is an eye-opener as it presents the idea of a medical system that is ecofriendly, biofriendly and is taking care of the human health with an equal care to the surrounding environment.

## 2. Early Model of Practicing Sustainable Medicine: Veterinary Science in Ayurveda

Veterinary science has an early mention in some of the most ancient literature of India. Atharvaveda, the progenitor of Ayurveda provides significant information about ailments of animals and their cure through herbal medicines. Ancient Indian history is full of events and incidences pertaining to the importance of livestock in then society. Many legends and myths of traditional Indian culture have been found associated with plants and animals building and reinforcing the idea of world as a family (*Vasudhaiva Kutumbakam*) where plants animals and human beings were considered with an equal importance. This would be surprising to many who are not aware of Indian culture that most of the legends in Hindu mythology have been made associated with some plants and or some animal as their representatives. This was probably to endorse importance to these creatures among the followers of the deity. Earlier India probably has lived a life concentric with religion at its center and the cow (and other animals) occupying an important place, not only merely on their nutritive lacto grounds, but also for cultural ethos [4].

Ayurveda has many mentions about the diseases of animals and their cures. In Charaka samhita with reference to Jwara (pyrexia) this is said that pyrexia affects every living being may it be human or animal or even insects. In earlier periods, when Ayurveda was supposed to be in full-blown practice, there were specific branches of veterinary Ayurveda dealing with different species of animals and their disease. The science was possibly more evolved and specialized as is indicated by various divisions of veterinary Ayurveda. The Gautam Samhita, the Ashva Ayurveda and Hastya Ayurveda are the ancient treatises on animal science available till now. Palakapya, an ultimate authority on elephant medicine belonged to the Rigvedic period 2000–4000 BC. He wrote Hastya Ayurveda dealing with elephant medicine and dedicated this to Lord Ganesha a deity with a human body and elephant head. Elephant medicine and surgery was further divided into four parts by Palakapya, viz, Maha Rogsthan or major diseases, Ksudra Roga Sthan or minor diseases, Shalya Sthan or surgery and Chikitsa Sthana or materia medica diet and Hygiene. He classified various ailments of elephants into: Adhyatmika (physical) and Agantuka (accidental or incidental); causes of ailments were classified as Manasa (caused by mental reasons) and Dosaja caused by disturbance of body humors—Vata, Pitta and Kapha. Hastya Ayurveda also mentions about anatomy of elephant, treatment of different kinds of diseases, training of elephant and also classification of elephants on the basis of a number of physical and trait characteristics. Shalihotra (2350 BC) was probably the first known veterinarian of the world and the father of Indian veterinary sciences.

## 3. The Paradigm: Using Veterinary Science for Human Health

Ancient literature of Ayurveda is full of medicines or formulations made of animal products. Substances, which are used in medicine and are derived from animals, are grouped under the head of Jangama dravya (material derived from animals). There are hundreds of such formulations in Ayurveda, which utilize a variety of animal products. These animal products are much diverse in their habitat of origin and comprise from marine, aquatic, terrestrial or avian species. In terrestrial animals, the products from wild as well as from domestic animals are used in medicine. Among a wide variety of products derived from animals and used in medicine, commonly utilized substances are honey, milk and its derivatives, bile, fat, bone marrow, blood, flesh, feces, urine, skin, semen, ligaments, bones, shell, horn and feathers [5]. Along with many usages of animal substances as medicine, Ayurveda also have developed certain ways of involving animals in human health care without causing harm to them. These approaches are unique to Ayurveda and require their revalidation in view of current science to establish the concept of sustainable medicine where the resources are not exploited for their usage to the extent leading to their extinction.

### 3.1. Animal Usage in Human Medicine

There are some very important sustainable medicinal usages of animals as mentioned in ancient text of Ayurveda. Some most notable among them are follows.

#### 3.1.1. Use of Milk from Cow That Is Fed upon Specific Leaves

Charaka samhita devotes a complete chapter (Mashparna Bhritiya) describing the quality of cow milk, which is fed upon either of leaves of Arjuna (Terminalia arjuna), Mash (Phaseolus mungo Linn.) leaves or beans or on Ikshu (Saccarum officinarum Linn.). The milk of a red or black cow, which is fed upon either of these substances, is considered as rejuvenator and aphrodisiac. This chapter further explains many other such preparations with similar medicinal properties and where this milk is used as a base component [6].

#### 3.1.2. Goat Acquaintance (Chag Seva Krama)

An acquaintance with goats has been appreciated as a remedial measure for tubercular patients. This acquaintance involves living between goats, and using their milk and its derivatives [7] for food.

#### 3.1.3. Use of Animal Derivatives That Can Be Acquired without Killing Them

Bhava prakasha, treatise on Ayurvedic materia medica has dealt in detail with the sustainable animal products that can be used in medicine [8]. This was grouped as milk and its derivatives, urine and madhu (honey). Milk is considered as a rejuvenator in Ayurveda and as many as eight genus of animals are described for the medicinal properties of their milk and its derivatives. These described genera are cow, buffalo, sheep, goat, camel, horse, elephant and human. Urine has also been given much importance in Ayurveda. Apart from human urine, cow urine is much praised for it medicinal properties. Medicinal usage of cow urine are extensively searched and scientifically endorsed [4].

### 3.2. Use of Animals and Their Products in Ayurvedic Surgery

There are not too many references of animal products used in surgery as described in Ayurveda. However, animal behavior, morphology and their habitat seems to have influenced Ayurvedic surgery to a larger extent. Most of the surgical instruments described in Ayurveda are inspired for their shape and functions on real time observation of animals and birds [9]. Forceps (Swastic Yantra) have specially been elaborated for their resemblance with mouth and beaks of different animal and birds and these are used for removal of foreign bodies embedded at different locations in a patient.

#### 3.2.1. Use of Absorbable Suture in Surgery

Use of self-absorbable and biodegradable sutures for internal surgery is considered as a milestone in modern surgical practice. Very surprisingly, the idea was found well conceived and practiced in Ayurveda as it was mentioned in Sushruta Samhita. In cases of intestinal perforation, after laparotomy, the perforation was to be identified, approximated and was to be exposed to black ants with their pincers like mouth appendages. Once the intestinal walls are approximated and gripped by these appendages, they were to be cut from the body of insect [10]. This was possibly the first ever example in the world history where a self-absorbable and biodegradable material was used in intestinal surgery. The body of insects is largely made of chitin, which is a complex protein carbohydrate material of biological origin and is a slowly degradable substance.

#### 3.2.2. Leech as a Mean of Blood Letting

Bloodletting is one of the most ancient ways of dealing with blood born diseases. This is not only described in ancient Ayurvedic texts but has also been found in practice of TCM. Among several ways through which the blood may be let out, leech is considered as the most sophisticated and softest way of doing the same. There have been a number of conditions where a blood letting through leech is advised. Important among them are disease of skin, tumors, nonhealing ulcers and thromboembolic condition of the blood vessels. The leech science was considerably developed in ancient India as is evident through description of multiple varieties of leeches and their elaborate methods of application [11].

### 3.3. Current Status of Use of Animals for Human Medicine

Use of animals and their derivatives as a part of the medicine meant for human use or as a part of the experimental and exploratory studies meant for evolving new researches supposed to be used is solving many of the still unresolved mysteries of human biology has largely been criticized on human, ethical and environmental grounds. Unscrupulous usage of animal products in TM has led to many undesirable consequences including illegal trafficking of animal products. Pocking of animals for their medicinally important parts has brought many of the wild species under the red data book, for a possibility of their extinction. Many genera and species of wild animals have been considered at the brim of extinction as a consequence of overexploitation either of their own or of their habitat. Worst sufferers of illegal trafficking for animal derivatives are rhinoceros for horn, reindeer for antlers, elephant for tusk, tiger for bone, peacock for feathers and musk deer for musk. TCM has largely been criticized by the world community for its unscrupulous use of animal products in many of its formulations.

The resultant threat upon wild animals was well perceived by international community. IUCN (International Union for Conservation of Nature and Natural Resources) maintained an international list called as red data book, which contains the species under perceived risk [12]. As a remedial measure many countries around the world have banned the formulations, which contain animal parts or the products.

Exploratory and experimental studies involving animals are criticized on account of cruelty issues. Animals under the experimental studies are often kept in stress conditions, are exposed to pain and different pharmacological products, and also to diseases artificially and are finally sacrificed to have their tissues studied. Though there are not the issues related to their extinction, there are issues regarding the grade of stress, which is given to them by human being for their own benefits.

## 4. Bioprospecting: Ayurvedic Approach

Out of many applications made of animals and their products, the most convincing and appealing is the use of products, which can be obtained without killing an animal. Pancha-Gavya therapy of Ayurveda, which utilizes five components obtained by cow (milk, curd, ghrita (milk butter), urine and dung) is a notable example of this approach. Cow urine has extensively been examined for its biopurificatory properties. It is used in almost every purification process of Ayurveda involving herbs or minerals. Significant reduction in the toxic properties of Vatsanabha (aconite) is observed when this is dipped in cow urine or milk [13]. Cow urine has also been found to increase phagocytosis by macrophages and thus is sought helpful in prevention and control of bacterial infections. Besides this, cow urine has antioxidant property which protects DNA damage due to mitomycin-C induced chromosomal aberrations [4]. Recently the cow urine has been granted US Patents (No. 6 896 907 and 6 410 059) for its medicinal properties, particularly for its use along with antibiotics for the control of bacterial infection and fight against cancers. Through extensive research studies a cow urine distillate fraction, popularly known as “ark”, has been identified as a bioenhancer of the activities of commonly used antibiotics, antifungal and anticancer drugs. Thus, it can promote and augment the bioactivity, bioavailability or the uptake of drugs in combination therapy and reduce the dose and duration of treatment. These milestone achievements highlight the beneficial role of cow urine in treating bacterial infections and cancers and that cow urine enhances the efficacy and potency of therapeutic drugs. During the past few years, cow urine therapy has provided promising and authentic results for the treatment of cancer, a deadly malady, which is being faced by the mankind and the incidences of which are ever increasing in the current scenario of changed lifestyle and food habits along with exposure to predisposing factors of carcinogens, such as tobacco chewing, smoking, alcohol intake, environmental pollutants, occupational health hazards, and so forth. Anticancer potenial of cow urine therapy has been reflected by several case reports, success stories and practical feed back of patients for the treatment of cancer. Cow urine enhances the immunocompetence and improves general health of an individual; prevent the free radicals formation and act as antiaging factor; reduces apoptosis in lymphocytes and helps them to survive; and efficiently repairs the damaged DNA, thus is effective for the cancer therapy [14].

Another facet of the sustainable use of animals in human health and disease management is through biotransformation. Animals, if fed on some specific ingredients in their diet are bound to have positive or negative impacts of the same, which can be reflected through the biochemical analysis of their secretions. This approach is fundamental to experimental and exploratory pharmacology, which eventually forces millions of pity animals to loose their lives as an offering to these experiments. Surprisingly, the concept was found in use in Ayurveda centuries before the advent of the modern and systematic studies in human biology although in a complete paradox. Animals if fed with the plants of certain pharmacological properties will have these properties reflected in their secretions. As per the pharmacokinetic principles, we are aware that how a drug moves in our body and how this gets eliminated. Pharmacodynamics also gives us the idea about possible interaction of these drugs with that of body tissue and secretions. There are many drugs that are secreted through the mother's milk. Ayurveda has beautifully utilized this concept of biotransformation by feeding the cow upon the certain plants with revitalizing properties. These inherent properties of the plants are possibly further enhanced in the animal body through xenobiosis and finally are reflected in the milk of the fed animal. Milk itself is considered as a revitalizer in Ayurveda and its properties are further synergized by addition of essence of the similar drugs. Charaka, the legendary Ayurvedic physician has dedicated a full chapter to this approach in his treatise Charaka samhita [6]. Ayurveda, in toto, presents the concept of sustainable medicine, which may be operating at multiple levels as effective preventive care, comprehensive primary care, better resource utilization and novel mechanism applications. All these mechanisms operate through various action plans and finally reach at a destination which is often called as ecologically sustainable medicine (Table 1).

## 5. Sustainability in Traditional Medicine: Where Is the Key?

The end of 20th century was earmarked with renewed interest and popularity of TM. This recent resurgence of natural therapies was mainly due to the realization of limitation of modern drugs in many conditions and also the observation of their increasing side effects. The ever escalating cost of the modern therapeutics and it's over dependence upon the diagnostic tools has also added to the grievances and thus the requirement of a safer, cheaper and at par (if not better) alternative system was largely felt. Traditional medical practices have sensed this gap and presented themselves as a dependable alternative at least initially. Now after the continuation of this trend for more than two decades, we are encountering different problems than we faced earlier. If the ongoing trends of escalating global demand for natural therapies are any indication, sooner or later we will find ourselves in the similar compartment where now the modern medicine is. Overused and overburdened with scientific principles (which often ignores the observational natural facts), TM is supposed to loose its face, which would have been a basic characteristic of the same. TM in principle is the medical practice, meant for local people, utilizing local resources and offering an opportunity to get healed without looking for many extravagant investigations. TM is sustainable medicine in its true sense as it takes care for the environment from where this is originating.

Environmentalist, educationist and activist globally with the similar idea have started thinking on this line through with their own version. Alternative methods for the exploratory and experimental practices involving animals are also being seriously looked for. The Johns Hopkins Center for Alternatives to Animal Testing (CAAT) is one such center that made itself dedicated to improving health for both people and animals. It promotes humane science by supporting the creation, development, validation and use of alternatives to animals in research, product safety testing and education [15]. Reduction, replacement and refinement (three R's) are the conventional ways of thinking about how to improve upon the existing situation of use of animals in medical science [16]. Now this seems to be our turn to look more seriously for the sustainability of our resources of TM, if we are willing to continue our contributions in bringing affordable health care for the generations to come. Use of veterinary science in Ayurveda gives us a lead to think upon some practical methods of its implementation. Bioprospecting in general and as is practiced in Ayurveda with respect to the usage of animal or plants could be the ultimate key, which can ensure a sustainable biodiversity till our future generations.

## 6. Perspectives

This review article has discussed the idea of sustainability in traditional medical practice of ancient periods and also attempted to explore relevance to current practice despite the absence of historical revelations. Speculations have been regarded as the progenitor of new researches in science. Any fundamental that is now regarded as a universal principle probably began as a speculation simply during the process of its primary formation. Thus, speculations are usually regarded highly and are considered as important for the growth of science. This review stressed the requirement of sustainability in TM where the resources are limited and are not rapidly renewable. An ignorance of this requirement will likely be detrimental to the health of TM in general but to the global environment as well. Conceptualization concerning sustainability that is not practiced is speculation and based upon experiences and trends; speculation must be respected for the good of global community.

The idea of sustainability in TM can well be traced through different cultures and societies with different notions [17–19]. Bioprospecting which is recently promoted as the most potential sector of pharmacological breakthroughs meant for human utilization conceives (i) chemical synthesis of the compounds, (ii) cultivation and (iii) the production of secondary metabolites in bioreactors as the method to deal with the sustainability issue in regards to the substrates derived from living beings [20, 21]. This approach, however, seems appropriate to less evolved animals (invertebrates) or plants (algae, fungi and bryophytes) only where reproductions rates are higher and so there is a rapid replication of their biomass in a short span of time. The same approach if applied to highly evolved plants or animals is bound to have its consequences in the terms of technological dependency, higher cost and time consumption. A chemical synthesis, cultivation and secondary metabolite production, despite of their promising prospects, can never act as the substitution to the ease, accessibility and economicity inherent to the practice of TM, which acted crucially for their growth in concurrent periods. Cooper in some of his recent writings [22, 23] has nicely outlined the beauty of CAM by saying “CAM is organismic, inclusive and not exclusive. This organismic approach involving the cells, tissues, organs and the molecules that they synthesize and secrete has fostered and indeed uncovered an incredible systemic amalgam, discovering almost daily an infinite array of new connections and interconnections, revealing ever more minute complexities almost to the point of incomprehension—as vast as the universe”. The most essential part of this organismic approach, inherent to every traditional medical system is the consideration of every living being as a cohabitant of the earth with equal respect to them in every sense. This approach does not asks for any need of exploitation or renaissance of the natural resources for trivial material benefits but instead asks for the grant of a small fraction from the eternity for the good of humanity. This is biorespecting and not the bioprospecting, which possibly represents traditional medical practice in a more closure way.

## Figures and Tables

**Table 1:** Sustainable TM: clue from ayurveda.

	Mechanism	Mode of action	Effect
1	Better preventive care	Life style modification	Less requirement of curative care leading to less burden upon resources
2	Comprehensive primary care	Promotion of home remedies, impowerment of primary care system through use of local resources	Less dependability upon curative care. Less resource utilization
3	Sustainable resource utilization	Resource utilization as per the actual need and not for the commercial gains	Less consumption of natural resources
4	Novel mechanism application	Application of xenobiosis, biotransformation and other mechanisms to improve effects	Reduced dose requirements requiring lesser resource consumption
